# 

**Published:** 1897-06

**Authors:** 


					﻿Vol. XVII.
JUNE, 1897.
NO. 6?
JBHE
flOMtEOPATHlC pHY^lClAJfl,
A Monthly Journal of Homoeopathic Materia Medica
and Clinical Medicine.
"Be it a freeman whom truth makes free, and all are slaves betide."
"Seek the Truth: come whence it may, cost what it will."
EDITED AND PUBLISHED BY
WALTER M. JAMES, M. D.
PHILADELPHIA :
□STo. 1231 LOCUST STREET.
ALFRED HEATH & CO., Sole Agents for Great Britain,
114 Ebury Street, Eaton Square.
Yearly Subscription, $2.50, invariably in advance. Single numbers, 25 els.
Entered at the Philadelphia Pc.t-OIBce aa Second-Claee Mall Matter.
				

